# Crystal structure and Hirshfeld surface analysis of phen­yl(5,7,8a-triphenyl-1,2,3,7,8,8a-hexa­hydro­imidazo[1,2-*a*]pyridin-6-yl)methanone with an unknown solvent

**DOI:** 10.1107/S2056989020009871

**Published:** 2020-07-24

**Authors:** Farid N. Naghiyev, Gunay Z. Mammadova, Ibrahim G. Mamedov, Afet T. Huseynova, Sevim Türktekin Çelikesir, Mehmet Akkurt, Anzurat A. Akobirshoeva

**Affiliations:** a Organic Chemistry Department, Baku State University, Z. Xalilov str. 23, Az, 1148 Baku, Azerbaijan; b Department of Physics, Faculty of Sciences, Erciyes University, 38039 Kayseri, Turkey; c Academy of Science of the Republic of Tadzhikistan, Kh. Yu. Yusufbekov Pamir Biological Institute, 1 Kholdorova St, Khorog 736002, Gbao, Tajikistan

**Keywords:** crystal structure, cyclo­addition products, hexa­hydro­imidazo[1,2-*a*]pyridine ring, SQUEEZE

## Abstract

In the title compound, C_32_H_28_N_2_O, the imidazolidine and pyridine rings of the central hexa­hydro­imidazo[1,2-*a*]pyridine ring system adopt envelope and screw-boat conformations, respectively. The mol­ecule exhibits two weak intra­molecular π–π inter­actions between phenyl rings. In the crystal, mol­ecules are linked *via* pairs of C—H⋯ O hydrogen bonds, forming inversion dimers. The dimers are further linked by pairs of C—H⋯π inter­actions, forming infinite chains along the *c*-axis direction. A Hirshfeld surface analysis indicates that the most important contributions to the crystal packing are from H⋯H (73.4%), C⋯H/H⋯C (18.8%) and O⋯H/H⋯O (5.7%) contacts. The contribution of some disordered solvent to the scattering was removed using the SQUEEZE routine [Spek (2015 ▸). *Acta Cryst*. C**71**, 9–18] in *PLATON*. The solvent contribution was not included in the reported mol­ecular weight and density.

## Abstract 2

The crystal exhibits weak intra­molecular π–π inter­actions between the phenyl rings. In the crystal, mol­ecules are linked *via* pairs of C—H⋯ O, forming inversion dimers. The dimers are further linked by pairs of C—H⋯π inter­actions, forming infinite chains along the *c*-axis direction.

## Chemical context   

Carbon–carbon and carbon–heteroatom bond-forming reactions are the most powerful and fundamental tools in synthetic organic chemistry. These synthetic approaches have successfully found applications in the construction of carbo- and heterocyclic ring systems (Khalilov *et al.*, 2011 ▸; Yin *et al.*, 2020 ▸). The use of nitro­gen as the bridgehead atom is being assessed extensively. Bridgehead nitro­gen heterocycles comprising imidazole rings are prevalent structural motifs in many compounds having applications in medicinal chemistry, coordination chemistry and material science (Afkhami *et al.*, 2017 ▸; Mahmoudi *et al.*, 2017*a* ▸,*b* ▸; Mahmudov *et al.*, 2019 ▸, 2020 ▸). Various imidazo[1,2-*a*]pyridine moieties are included in synthetic drugs, such as alpidem, olprinone, saripidem, necopidem, miroprofen, zolimidine and zolpidem, which have already found use in medicinal practice. On the other hand, the imidazo[1,2-*a*]pyridine motif is also found in a series of natural products, such as oxaline and neoxaline (Koizumi *et al.*, 2004 ▸). As a result of the considerable inter­est to this field, there have been significant developments in the synthesis of imidazo[1,2-*a*]pyridine derivatives. In the framework of our ongoing structural studies (Akkurt *et al.*, 2018 ▸; Khalilov *et al.*, 2019 ▸), we report herein the crystal structure and Hirshfeld surface analysis of the title compound.
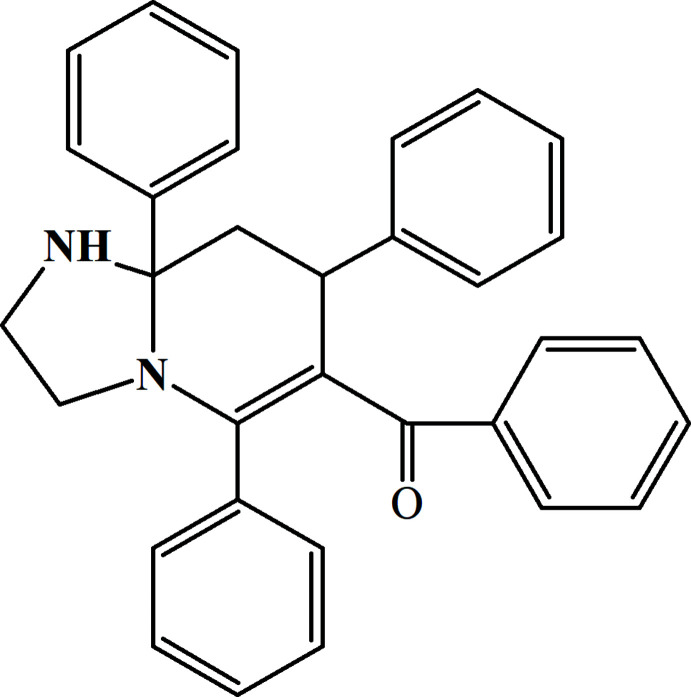


## Structural commentary   

**Figure 1:**
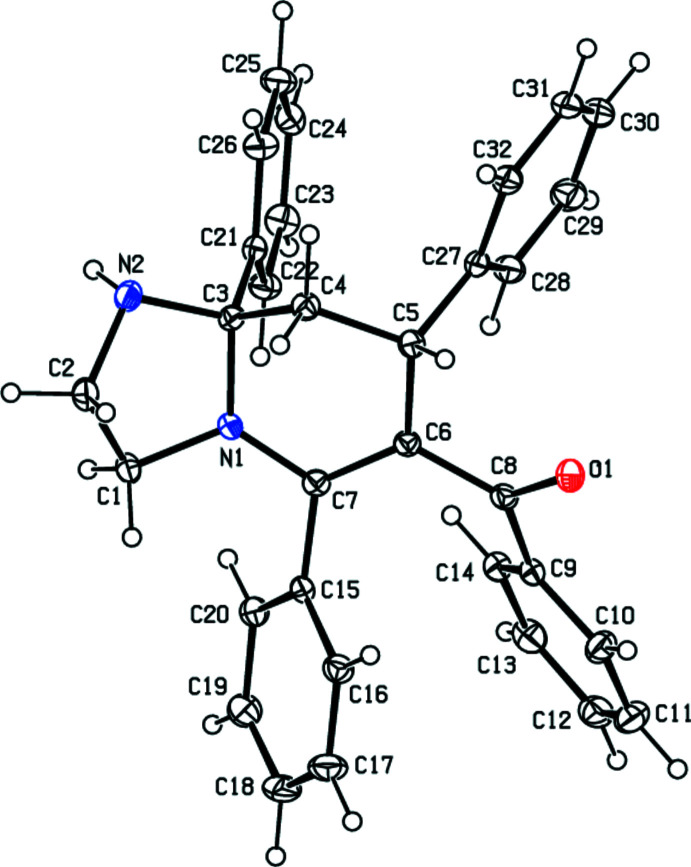
The mol­ecular structure of the title compound, with displacement ellipsoids drawn at the 30% probability level.

In the title compound (Fig. 1 ▸), the imidazolidine ring (N1/C1/C2/N2/C3) of the central hexa­hydro­imidazo[1,2-*a*]pyridine ring system (N1/C1/C2/N2/C3–C7) adopts an envelope conformation with atom C2 as the flap lying 0.222 (2) Å from the mean plane of the remaining four atoms, while the pyridine ring (N1/C3–C7) is puckered with the puckering parameters *Q*_T_ = 0.4970 (15) Å, *θ* = 62.27 (17)° and *φ* = 96.49 (19)°. The dihedral angles between phenyl rings are *A*/*B* = 34.51 (8), *A*/*C* = 48.27 (8), *A*/*D* = 74.89 (8), *B*/*C* = 37.27 (8), *B*/*D* = 56.29 (8) and *C*/*D* = 26.72 (8)°, where *A*, *B*, *C* and *D* are the phenyl rings C9–C14, C15–C20, C21–C26 and C27–C32, respectively. The *A*, *B*, *C* and *D* ring planes are inclined to the central hexa­hydro­imidazo[1,2-*a*]pyridine ring system, making dihedral angles of 60.24 (7), 61.73 (7), 81.91 (7) and 63.08 (7)°, respectively, with the mean plane of the central ring system. There are two weak intra­molecular π–π inter­actions [*Cg*3⋯*Cg*4 = 3.7628 (11) Å and *Cg*5⋯*Cg*6 = 3.9822 (10) Å; *Cg*3, *Cg*4, *Cg*5 and *Cg*6 are the centroids of rings *A*, *B*, *C* and *D*, respectively].

## Supra­molecular features and Hirshfeld surface analysis   

**Figure 2:**
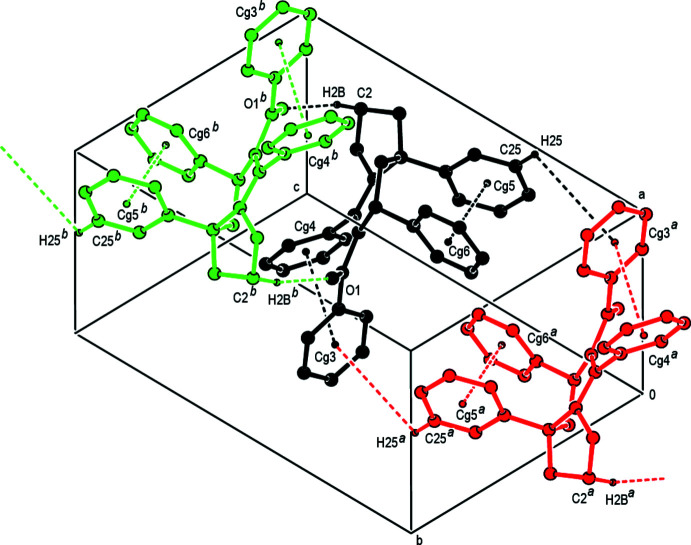
The crystal packing of the title compound. Dashed lines indicate C—H⋯O, C—H⋯π and π–π stacking inter­actions. *Cg*3, *Cg*4, *Cg*5 and *Cg*6 are the centroids of the C9–C14, C15–C20, C21–C26 and C27–C32 phenyl rings, respectively. [Symmetry codes: (*a*) −*x*, −*y*, −*z* + 1; (*b*) −*x*, −*y*, −*z* + 2.]

In the crystal, mol­ecules are linked *via* pairs of C—H⋯ O hydrogen bonds, forming inversion dimers. The dimers are further linked by pairs of C—H⋯π inter­actions, forming an infinite chain along the *c*-axis direction (Table 1 ▸ and Fig. 2 ▸).

**Figure 3:**
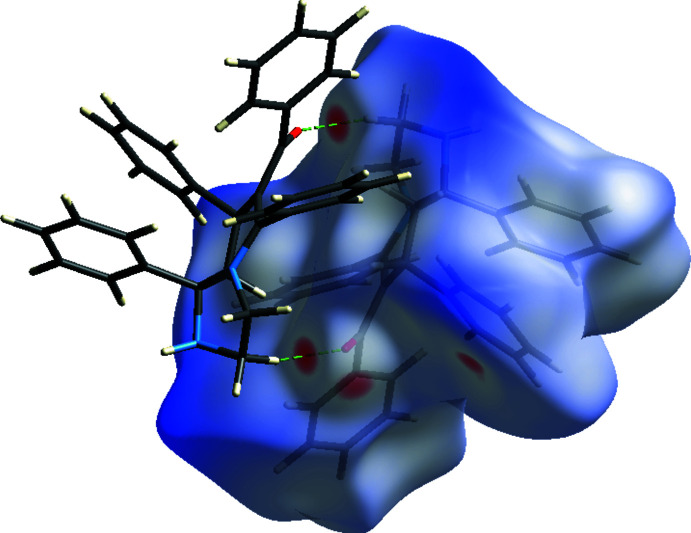
A view of the Hirshfeld surface of the title compound plotted over *d*_norm_, showing the C—H⋯O inter­actions.

**Figure 4:**
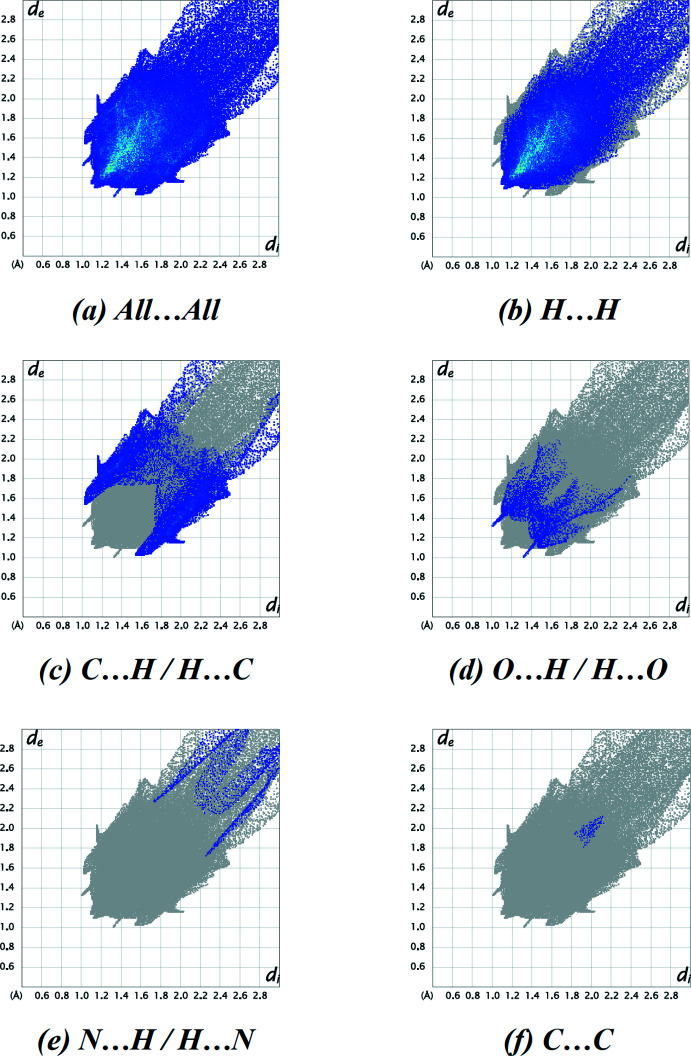
(*a*) A full two-dimensional fingerprint plot for the title compound, together with those delineated into (*b*) H⋯H, (*c*) C⋯H/H⋯C, (*d*) O⋯H/H⋯O, (*e*) N⋯H/H⋯N and (*f*) C⋯C contacts.

In order to obtain further insight into the inter­molecular inter­actions, we used *Crystal Explorer* (Turner *et al.*, 2017 ▸). The Hirshfeld surface of the title compound mapped over *d*_norm_ is depicted in Fig. 3 ▸, where the red regions are apparent around atom O1, which participates in the C—H⋯O inter­actions (Table 1 ▸). The fingerprint plots (Fig. 4 ▸) show that the largest contribution to the overall crystal packing is from H⋯H contacts (73.4%). The second largest percentage (18.8%) can be attributed to C⋯H/H⋯C contacts, which correlate with the C—H⋯π inter­actions. O⋯H/H⋯O contacts (5.7%), which correlate with the C—H⋯O inter­actions, provide another significant contribution to the Hirshfeld surface. Other contributions include N⋯H/H⋯N (1.9%) and C⋯C (0.2%). The removal of the contribution of the disordered solvent to the scattering using the SQUEEZE routine of *PLATON* may be responsible for a small change in the given percentage contributions.

## Database survey   

A search in the Cambridge Structural Database (CSD, Version 5.41, updated to March 2020; Groom *et al.*, 2016 ▸) gave three hits for the 1,2,3,7,8,8a-hexa­hydro­imidazo[1,2-*a*]pyridine moiety, *viz*. 5,7,8a-triphenyl-1,2,3,7,8,8*a*-hexa­hydro­imidazo[1,2-*a*]pyridine (KICJUE; Alvim *et al.*, 2018 ▸), 7-(4-bromo­phen­yl)-5,8a-diphenyl-1,2,3,7,8,8*a*-hexa­hydro­imidazo[1,2-*a*]pyridine (TEZJOZ; Wang *et al.*, 2013 ▸) and 8-benz­yloxy-8a-methyl-1,2,3,7,8,8a-hexa­hydro­imidazo[1,2-*a*]pyridin-7-one monohydrate (YUYREP; Wireko *et al.*, 1995 ▸). In KICJUE, single crystal X-ray analysis confirmed the *trans* derivative as the only isomer. The structure of TEZJOZ shows that the aromatic ring of the aldehyde is on the other plane of the ketone in the purposed mechanism for the reaction. In the crystal of YUYREP, each water mol­ecule bridges two mol­ecules of the compound, hydrogen bonding with the carbonyl O atom of one mol­ecule [O⋯O*W* = 2.796 (4) Å] and with the N atom of the other [N⋯O*W* = 2.903 (4) Å]. The methyl group at the bridgehead is axially located in a *trans* position with respect to the bulky benz­yloxy group. The pyridone ring assumes a slightly distorted half-chair conformation.

## Synthesis and crystallization   

To a solution of 2-benzoyl-1,3,5-tri­phenyl­pentane-1,5-dione (3.5 mmol) in ethanol (35 ml) was added ethyl­enedi­amine (3.7 mmol) and 5 drops of concentrated HCl. The mixture was stirred at room temperature for 15 min, then refluxed for 4 h and cooled down to room temperature. The reaction product precipitated from the reaction mixture as colourless single crystals, which were collected by filtration and purified by recrystallization from ethanol (yield 76%; m.p. 465–466 K).

^1^H NMR (300 MHz, DMSO-*d*_6_): *δ* 2.28 (*dd*, 2H, CH_2_N), 2.77 (*dd*, 2H, CH_2_N), 3.02 (*t*, 1H, CH), 3.41–3.63 (*dd*, 2H, CH_2_), 5.34 (*s*, 1H, NH), 6.82–7.78 (*m*, 20H, 4Ar-H). ^13^C NMR (75 MHz, DMSO-*d*_6_): *δ* 37.63, 45.55, 48.71, 48.98, 75.80, 125.99, 126.72, 127.53, 128.06, 128.18, 128.43, 128.54, 128.99, 133.34, 136.99, 145.47, 146.49, 170.71, 199.38.

## Refinement details   

Crystal data, data collection and structure refinement details are summarized in Table 2 ▸. The N-bound H atom was located in a difference-Fourier map and refined freely [N2—H2*N* = 0.908 (16) Å]. The remaining H atoms were placed in calculated positions (C—H = 0.95–1.00 Å) and allowed to ride on their carrier atoms, with *U*_iso_ = 1.2*U*_eq_(C). The residual electron density was difficult to model and therefore the SQUEEZE routine (Spek, 2015 ▸) in *PLATON* (Spek, 2020 ▸) was used to remove the contribution of the electron density in the solvent region from the intensity data and the solvent-free model was employed for the final refinement. The solvent formula mass and unit-cell characteristics were not taken into account during refinement. The cavity of volume *ca* 119 Å^3^ (*ca* 9.4% of the unit-cell volume) contains approximately 28 electrons.

## Supplementary Material

Crystal structure: contains datablock(s) I. DOI: 10.1107/S2056989020009871/is5547sup1.cif

Structure factors: contains datablock(s) I. DOI: 10.1107/S2056989020009871/is5547Isup2.hkl

Supporting information file. DOI: 10.1107/S2056989020009871/is5547Isup3.cml

CCDC reference: 2017791

Additional supporting information:  crystallographic information; 3D view; checkCIF report

## Figures and Tables

**Table 1:** Hydrogen-bond geometry (Å, °) *Cg*3 is the centroid of the C9–C14 phenyl ring.

*D*—H⋯*A*	*D*—H	H⋯*A*	*D*⋯*A*	*D*—H⋯*A*
C2—H2*B*⋯O1^i^	0.99	2.43	3.4084 (19)	168
C24—H24⋯*Cg*3^ii^	0.95	2.83	3.5886 (18)	138

Symmetry codes: (i) 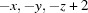; (ii) 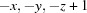.

**Table 2:** Experimental details

Crystal data	
Chemical formula	C_32_H_28_N_2_O
*M*_r_	456.56
Crystal system, space group	Triclinic, *P* 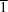
Temperature (K)	150
*a*, *b*, *c* (Å)	8.7807 (9), 11.9566 (12), 12.9121 (13)
α, β, γ (°)	77.982 (1), 78.711 (1), 75.612 (1)
*V* (Å^3^)	1269.4 (2)
*Z*	2
Radiation type	Mo *K*α
μ (mm^−1^)	0.07
Crystal size (mm)	0.28 × 0.25 × 0.23

Data collection	
Diffractometer	Bruker P4
Absorption correction	Multi-scan (*SADABS*; Sheldrick, 1996 ▸)
*T*_min_, *T*_max_	0.980, 0.984
No. of measured, independent and observed [*I* > 2σ(*I*)] reflections	16061, 6472, 4328
*R*_int_	0.042
(sin θ/λ)_max_ (Å^−1^)	0.695

Refinement	
*R*[*F* ^2^ > 2σ(*F* ^2^)], *wR*(*F* ^2^), *S*	0.051, 0.117, 1.03
No. of reflections	6472
No. of parameters	320
H-atom treatment	H atoms treated by a mixture of independent and constrained refinement
Δρ_max_, Δρ_min_ (e Å^−3^)	0.25, −0.23

Computer programs: *XSCANS* (Bruker, 2007 ▸), *SHELXTL* (Sheldrick, 2008 ▸), *SHELXT2014/5* (Sheldrick, 2015*a* ▸), *SHELXL2018/3* (Sheldrick, 2015*b* ▸), *ORTEP-3 for Windows* (Farrugia, 2012 ▸) and *PLATON* (Spek, 2020 ▸).

## supplementary crystallographic information

### Crystal data

**Table:**

C_32_H_28_N_2_O	*Z* = 2
*M**_r_* = 456.56	*F*(000) = 484
Triclinic, *P*1	*D*_x_ = 1.195 Mg m^−^^3^
*a* = 8.7807 (9) Å	Mo *K*α radiation, λ = 0.71073 Å
*b* = 11.9566 (12) Å	Cell parameters from 2538 reflections
*c* = 12.9121 (13) Å	θ = 2.2–24.2°
α = 77.982 (1)°	µ = 0.07 mm^−^^1^
β = 78.711 (1)°	*T* = 150 K
γ = 75.612 (1)°	Prism, colourless
*V* = 1269.4 (2) Å^3^	0.28 × 0.25 × 0.23 mm

### Data collection

**Table:**

Bruker P4 diffractometer	6472 independent reflections
Radiation source: sealed tube	4328 reflections with *I* > 2σ(*I*)
Graphite monochromator	*R*_int_ = 0.042
ω scans	θ_max_ = 29.6°, θ_min_ = 1.8°
Absorption correction: multi-scan (SADABS; Sheldrick, 1996)	*h* = −12→12
*T*_min_ = 0.980, *T*_max_ = 0.984	*k* = −16→16
16061 measured reflections	*l* = −17→17

### Refinement

**Table:**

Refinement on *F*^2^	0 restraints
Least-squares matrix: full	Hydrogen site location: mixed
*R*[*F*^2^ > 2σ(*F*^2^)] = 0.051	H atoms treated by a mixture of independent and constrained refinement
*wR*(*F*^2^) = 0.117	*w* = 1/[σ^2^(*F*_o_^2^) + (0.0474*P*)^2^ + 0.0165*P*] where *P* = (*F*_o_^2^ + 2*F*_c_^2^)/3
*S* = 1.03	(Δ/σ)_max_ < 0.001
6472 reflections	Δρ_max_ = 0.25 e Å^−^^3^
320 parameters	Δρ_min_ = −0.23 e Å^−^^3^

### Special details

**Table:**

Geometry. All esds (except the esd in the dihedral angle between two l.s. planes) are estimated using the full covariance matrix. The cell esds are taken into account individually in the estimation of esds in distances, angles and torsion angles; correlations between esds in cell parameters are only used when they are defined by crystal symmetry. An approximate (isotropic) treatment of cell esds is used for estimating esds involving l.s. planes.

### Fractional atomic coordinates and isotropic or equivalent isotropic displacement parameters (Å^2^)

**Table:**

	*x*	*y*	*z*	*U*_iso_*/*U*_eq_	
C1	0.44939 (17)	0.02866 (13)	0.83139 (12)	0.0254 (3)	
H1A	0.417532	0.092805	0.874131	0.031*	
H1B	0.532449	0.047058	0.770600	0.031*	
C2	0.50577 (18)	−0.08820 (13)	0.89972 (12)	0.0268 (3)	
H2A	0.620705	−0.101848	0.903228	0.032*	
H2B	0.446137	−0.091719	0.973388	0.032*	
C3	0.32476 (16)	−0.11911 (12)	0.80213 (11)	0.0207 (3)	
C4	0.18403 (17)	−0.15217 (12)	0.88356 (11)	0.0228 (3)	
H4A	0.188777	−0.236789	0.889250	0.027*	
H4B	0.193319	−0.138146	0.954753	0.027*	
C5	0.02321 (16)	−0.08252 (12)	0.85304 (11)	0.0210 (3)	
H5	−0.054074	−0.085719	0.921116	0.025*	
C6	0.02583 (16)	0.04591 (12)	0.81466 (11)	0.0195 (3)	
C7	0.16526 (16)	0.08404 (12)	0.80012 (11)	0.0198 (3)	
C8	−0.13267 (17)	0.12259 (12)	0.81093 (11)	0.0215 (3)	
C9	−0.16490 (17)	0.23969 (12)	0.74001 (12)	0.0229 (3)	
C10	−0.29651 (18)	0.32328 (14)	0.77432 (13)	0.0316 (4)	
H10	−0.360310	0.305622	0.841463	0.038*	
C11	−0.3348 (2)	0.43204 (14)	0.71104 (14)	0.0390 (4)	
H11	−0.423388	0.489185	0.735723	0.047*	
C12	−0.2450 (2)	0.45780 (14)	0.61222 (14)	0.0373 (4)	
H12	−0.271383	0.532550	0.569140	0.045*	
C13	−0.1165 (2)	0.37451 (14)	0.57624 (13)	0.0331 (4)	
H13	−0.055757	0.391481	0.507747	0.040*	
C14	−0.07625 (18)	0.26622 (13)	0.63998 (12)	0.0257 (3)	
H14	0.012783	0.209572	0.615064	0.031*	
C15	0.17191 (16)	0.20907 (12)	0.78945 (11)	0.0206 (3)	
C16	0.07779 (18)	0.27811 (13)	0.86290 (12)	0.0262 (3)	
H16	0.010893	0.244616	0.922039	0.031*	
C17	0.0815 (2)	0.39533 (14)	0.84988 (14)	0.0377 (4)	
H17	0.017135	0.441920	0.900193	0.045*	
C18	0.1783 (2)	0.44495 (14)	0.76422 (15)	0.0409 (4)	
H18	0.179183	0.525745	0.754894	0.049*	
C19	0.27404 (19)	0.37652 (14)	0.69207 (14)	0.0361 (4)	
H19	0.341594	0.410217	0.633493	0.043*	
C20	0.27169 (17)	0.25941 (13)	0.70494 (12)	0.0267 (3)	
H20	0.338866	0.212733	0.655604	0.032*	
C21	0.33725 (16)	−0.15426 (12)	0.69346 (11)	0.0211 (3)	
C22	0.34714 (18)	−0.07518 (13)	0.59878 (12)	0.0264 (3)	
H22	0.341812	0.004668	0.600857	0.032*	
C23	0.36476 (18)	−0.11116 (14)	0.50068 (12)	0.0306 (4)	
H23	0.371794	−0.055874	0.436337	0.037*	
C24	0.37210 (18)	−0.22659 (14)	0.49615 (13)	0.0297 (4)	
H24	0.382339	−0.251013	0.429194	0.036*	
C25	0.36436 (19)	−0.30625 (13)	0.59033 (13)	0.0322 (4)	
H25	0.370629	−0.386175	0.587958	0.039*	
C26	0.34759 (18)	−0.27082 (13)	0.68784 (12)	0.0292 (4)	
H26	0.343066	−0.326753	0.751869	0.035*	
C27	−0.03918 (16)	−0.13787 (12)	0.77793 (12)	0.0221 (3)	
C28	−0.05435 (18)	−0.08759 (13)	0.67303 (12)	0.0275 (3)	
H28	−0.024649	−0.014775	0.644757	0.033*	
C29	−0.1118 (2)	−0.14096 (15)	0.60841 (14)	0.0363 (4)	
H29	−0.120172	−0.104929	0.536272	0.044*	
C30	−0.15699 (19)	−0.24589 (15)	0.64757 (15)	0.0378 (4)	
H30	−0.197606	−0.281885	0.603040	0.045*	
C31	−0.14290 (18)	−0.29836 (14)	0.75188 (15)	0.0350 (4)	
H31	−0.172882	−0.371193	0.779443	0.042*	
C32	−0.08480 (18)	−0.24458 (13)	0.81661 (13)	0.0292 (4)	
H32	−0.075911	−0.281070	0.888577	0.035*	
N1	0.31103 (13)	0.00872 (10)	0.79395 (9)	0.0209 (3)	
N2	0.47413 (15)	−0.17512 (11)	0.84545 (11)	0.0263 (3)	
H2N	0.553 (2)	−0.1864 (13)	0.7890 (13)	0.033 (4)*	
O1	−0.24961 (12)	0.08722 (9)	0.86497 (8)	0.0286 (3)	

### Atomic displacement parameters (Å^2^)

**Table:**

	*U*^11^	*U*^22^	*U*^33^	*U*^12^	*U*^13^	*U*^23^
C1	0.0190 (8)	0.0297 (8)	0.0306 (8)	−0.0071 (6)	−0.0052 (6)	−0.0079 (7)
C2	0.0208 (8)	0.0331 (9)	0.0278 (8)	−0.0051 (7)	−0.0058 (6)	−0.0068 (7)
C3	0.0187 (7)	0.0172 (7)	0.0251 (8)	−0.0023 (6)	−0.0034 (6)	−0.0029 (6)
C4	0.0237 (8)	0.0212 (7)	0.0227 (8)	−0.0056 (6)	−0.0024 (6)	−0.0019 (6)
C5	0.0191 (7)	0.0217 (7)	0.0214 (7)	−0.0069 (6)	0.0006 (6)	−0.0027 (6)
C6	0.0199 (7)	0.0207 (7)	0.0186 (7)	−0.0049 (6)	−0.0011 (6)	−0.0055 (6)
C7	0.0214 (7)	0.0222 (7)	0.0161 (7)	−0.0049 (6)	−0.0008 (6)	−0.0056 (6)
C8	0.0209 (7)	0.0236 (7)	0.0222 (7)	−0.0060 (6)	−0.0010 (6)	−0.0095 (6)
C9	0.0197 (7)	0.0240 (8)	0.0279 (8)	−0.0047 (6)	−0.0063 (6)	−0.0083 (6)
C10	0.0263 (9)	0.0321 (9)	0.0346 (9)	0.0008 (7)	−0.0043 (7)	−0.0103 (7)
C11	0.0374 (10)	0.0297 (9)	0.0488 (11)	0.0051 (8)	−0.0142 (8)	−0.0123 (8)
C12	0.0440 (11)	0.0256 (9)	0.0463 (11)	−0.0066 (8)	−0.0222 (9)	−0.0016 (7)
C13	0.0378 (10)	0.0328 (9)	0.0314 (9)	−0.0127 (8)	−0.0096 (7)	−0.0013 (7)
C14	0.0241 (8)	0.0266 (8)	0.0282 (8)	−0.0055 (6)	−0.0058 (6)	−0.0068 (6)
C15	0.0185 (7)	0.0211 (7)	0.0244 (8)	−0.0057 (6)	−0.0061 (6)	−0.0040 (6)
C16	0.0271 (8)	0.0270 (8)	0.0264 (8)	−0.0075 (7)	−0.0023 (6)	−0.0080 (6)
C17	0.0416 (10)	0.0283 (9)	0.0455 (10)	−0.0085 (8)	0.0022 (8)	−0.0182 (8)
C18	0.0419 (11)	0.0203 (8)	0.0618 (12)	−0.0120 (7)	−0.0029 (9)	−0.0083 (8)
C19	0.0311 (9)	0.0282 (9)	0.0476 (11)	−0.0130 (7)	0.0008 (8)	−0.0013 (8)
C20	0.0228 (8)	0.0256 (8)	0.0316 (9)	−0.0072 (6)	0.0000 (6)	−0.0058 (7)
C21	0.0158 (7)	0.0230 (7)	0.0246 (8)	−0.0039 (6)	−0.0015 (6)	−0.0057 (6)
C22	0.0290 (8)	0.0226 (8)	0.0280 (8)	−0.0082 (6)	0.0001 (7)	−0.0059 (6)
C23	0.0326 (9)	0.0338 (9)	0.0241 (8)	−0.0094 (7)	0.0014 (7)	−0.0047 (7)
C24	0.0252 (8)	0.0390 (9)	0.0278 (8)	−0.0104 (7)	0.0016 (7)	−0.0134 (7)
C25	0.0357 (9)	0.0250 (8)	0.0381 (9)	−0.0093 (7)	−0.0001 (7)	−0.0120 (7)
C26	0.0346 (9)	0.0232 (8)	0.0288 (8)	−0.0061 (7)	−0.0020 (7)	−0.0050 (6)
C27	0.0158 (7)	0.0209 (7)	0.0295 (8)	−0.0034 (6)	0.0001 (6)	−0.0078 (6)
C28	0.0297 (9)	0.0244 (8)	0.0304 (9)	−0.0072 (7)	−0.0036 (7)	−0.0083 (6)
C29	0.0391 (10)	0.0367 (10)	0.0355 (9)	−0.0026 (8)	−0.0095 (8)	−0.0148 (8)
C30	0.0320 (9)	0.0373 (10)	0.0518 (11)	−0.0033 (8)	−0.0096 (8)	−0.0263 (9)
C31	0.0271 (9)	0.0233 (8)	0.0576 (12)	−0.0075 (7)	−0.0029 (8)	−0.0143 (8)
C32	0.0253 (8)	0.0228 (8)	0.0389 (9)	−0.0061 (6)	−0.0027 (7)	−0.0045 (7)
N1	0.0177 (6)	0.0212 (6)	0.0255 (6)	−0.0057 (5)	−0.0032 (5)	−0.0061 (5)
N2	0.0206 (7)	0.0289 (7)	0.0289 (7)	−0.0016 (5)	−0.0042 (6)	−0.0080 (6)
O1	0.0201 (5)	0.0320 (6)	0.0333 (6)	−0.0078 (5)	0.0015 (5)	−0.0069 (5)

### Geometric parameters (Å, º)

**Table:**

C1—N1	1.4794 (18)	C15—C20	1.3910 (19)
C1—C2	1.513 (2)	C15—C16	1.3943 (19)
C1—H1A	0.9900	C16—C17	1.384 (2)
C1—H1B	0.9900	C16—H16	0.9500
C2—N2	1.4736 (18)	C17—C18	1.381 (2)
C2—H2A	0.9900	C17—H17	0.9500
C2—H2B	0.9900	C18—C19	1.383 (2)
C3—N2	1.4752 (18)	C18—H18	0.9500
C3—N1	1.4859 (17)	C19—C20	1.380 (2)
C3—C21	1.5231 (19)	C19—H19	0.9500
C3—C4	1.5328 (18)	C20—H20	0.9500
C4—C5	1.530 (2)	C21—C22	1.384 (2)
C4—H4A	0.9900	C21—C26	1.3901 (19)
C4—H4B	0.9900	C22—C23	1.389 (2)
C5—C6	1.5166 (18)	C22—H22	0.9500
C5—C27	1.5279 (19)	C23—C24	1.379 (2)
C5—H5	1.0000	C23—H23	0.9500
C6—C7	1.3761 (19)	C24—C25	1.381 (2)
C6—C8	1.4675 (19)	C24—H24	0.9500
C7—N1	1.3685 (17)	C25—C26	1.380 (2)
C7—C15	1.4872 (18)	C25—H25	0.9500
C8—O1	1.2379 (16)	C26—H26	0.9500
C8—C9	1.500 (2)	C27—C28	1.380 (2)
C9—C14	1.390 (2)	C27—C32	1.3957 (19)
C9—C10	1.393 (2)	C28—C29	1.380 (2)
C10—C11	1.385 (2)	C28—H28	0.9500
C10—H10	0.9500	C29—C30	1.376 (2)
C11—C12	1.382 (2)	C29—H29	0.9500
C11—H11	0.9500	C30—C31	1.378 (2)
C12—C13	1.381 (2)	C30—H30	0.9500
C12—H12	0.9500	C31—C32	1.388 (2)
C13—C14	1.386 (2)	C31—H31	0.9500
C13—H13	0.9500	C32—H32	0.9500
C14—H14	0.9500	N2—H2N	0.908 (16)
			
N1—C1—C2	101.91 (11)	C16—C15—C7	120.90 (12)
N1—C1—H1A	111.4	C17—C16—C15	120.18 (14)
C2—C1—H1A	111.4	C17—C16—H16	119.9
N1—C1—H1B	111.4	C15—C16—H16	119.9
C2—C1—H1B	111.4	C18—C17—C16	120.41 (15)
H1A—C1—H1B	109.3	C18—C17—H17	119.8
N2—C2—C1	104.60 (12)	C16—C17—H17	119.8
N2—C2—H2A	110.8	C17—C18—C19	119.71 (15)
C1—C2—H2A	110.8	C17—C18—H18	120.1
N2—C2—H2B	110.8	C19—C18—H18	120.1
C1—C2—H2B	110.8	C20—C19—C18	120.19 (15)
H2A—C2—H2B	108.9	C20—C19—H19	119.9
N2—C3—N1	105.07 (11)	C18—C19—H19	119.9
N2—C3—C21	108.59 (11)	C19—C20—C15	120.63 (14)
N1—C3—C21	112.32 (11)	C19—C20—H20	119.7
N2—C3—C4	109.35 (11)	C15—C20—H20	119.7
N1—C3—C4	107.36 (11)	C22—C21—C26	118.31 (13)
C21—C3—C4	113.77 (11)	C22—C21—C3	122.27 (12)
C5—C4—C3	112.67 (11)	C26—C21—C3	119.33 (13)
C5—C4—H4A	109.1	C21—C22—C23	120.79 (14)
C3—C4—H4A	109.1	C21—C22—H22	119.6
C5—C4—H4B	109.1	C23—C22—H22	119.6
C3—C4—H4B	109.1	C24—C23—C22	120.40 (15)
H4A—C4—H4B	107.8	C24—C23—H23	119.8
C6—C5—C27	114.32 (12)	C22—C23—H23	119.8
C6—C5—C4	110.99 (11)	C23—C24—C25	119.08 (14)
C27—C5—C4	113.18 (11)	C23—C24—H24	120.5
C6—C5—H5	105.9	C25—C24—H24	120.5
C27—C5—H5	105.9	C26—C25—C24	120.62 (14)
C4—C5—H5	105.9	C26—C25—H25	119.7
C7—C6—C8	124.93 (12)	C24—C25—H25	119.7
C7—C6—C5	120.66 (12)	C25—C26—C21	120.79 (14)
C8—C6—C5	113.82 (12)	C25—C26—H26	119.6
N1—C7—C6	122.21 (12)	C21—C26—H26	119.6
N1—C7—C15	114.14 (12)	C28—C27—C32	117.59 (14)
C6—C7—C15	123.64 (12)	C28—C27—C5	123.52 (13)
O1—C8—C6	118.86 (13)	C32—C27—C5	118.89 (13)
O1—C8—C9	116.85 (13)	C29—C28—C27	121.33 (15)
C6—C8—C9	124.17 (12)	C29—C28—H28	119.3
C14—C9—C10	118.77 (14)	C27—C28—H28	119.3
C14—C9—C8	123.47 (13)	C30—C29—C28	120.55 (16)
C10—C9—C8	117.65 (13)	C30—C29—H29	119.7
C11—C10—C9	120.35 (15)	C28—C29—H29	119.7
C11—C10—H10	119.8	C29—C30—C31	119.46 (15)
C9—C10—H10	119.8	C29—C30—H30	120.3
C12—C11—C10	120.33 (16)	C31—C30—H30	120.3
C12—C11—H11	119.8	C30—C31—C32	119.85 (15)
C10—C11—H11	119.8	C30—C31—H31	120.1
C13—C12—C11	119.80 (16)	C32—C31—H31	120.1
C13—C12—H12	120.1	C31—C32—C27	121.21 (16)
C11—C12—H12	120.1	C31—C32—H32	119.4
C12—C13—C14	120.08 (16)	C27—C32—H32	119.4
C12—C13—H13	120.0	C7—N1—C1	123.36 (11)
C14—C13—H13	120.0	C7—N1—C3	120.74 (11)
C13—C14—C9	120.64 (14)	C1—N1—C3	109.49 (11)
C13—C14—H14	119.7	C2—N2—C3	105.65 (11)
C9—C14—H14	119.7	C2—N2—H2N	107.9 (10)
C20—C15—C16	118.84 (13)	C3—N2—H2N	107.7 (10)
C20—C15—C7	120.26 (12)		
			
N1—C1—C2—N2	34.54 (14)	N2—C3—C21—C22	−110.98 (15)
N2—C3—C4—C5	170.85 (11)	N1—C3—C21—C22	4.78 (18)
N1—C3—C4—C5	57.36 (15)	C4—C3—C21—C22	127.01 (14)
C21—C3—C4—C5	−67.56 (15)	N2—C3—C21—C26	65.54 (16)
C3—C4—C5—C6	−43.64 (15)	N1—C3—C21—C26	−178.70 (12)
C3—C4—C5—C27	86.46 (14)	C4—C3—C21—C26	−56.47 (17)
C27—C5—C6—C7	−122.71 (14)	C26—C21—C22—C23	0.9 (2)
C4—C5—C6—C7	6.78 (18)	C3—C21—C22—C23	177.43 (14)
C27—C5—C6—C8	65.67 (15)	C21—C22—C23—C24	0.2 (2)
C4—C5—C6—C8	−164.83 (11)	C22—C23—C24—C25	−1.1 (2)
C8—C6—C7—N1	−173.38 (13)	C23—C24—C25—C26	0.8 (2)
C5—C6—C7—N1	16.0 (2)	C24—C25—C26—C21	0.4 (2)
C8—C6—C7—C15	6.4 (2)	C22—C21—C26—C25	−1.2 (2)
C5—C6—C7—C15	−164.19 (12)	C3—C21—C26—C25	−177.83 (14)
C7—C6—C8—O1	−150.77 (14)	C6—C5—C27—C28	15.65 (19)
C5—C6—C8—O1	20.43 (18)	C4—C5—C27—C28	−112.74 (15)
C7—C6—C8—C9	33.3 (2)	C6—C5—C27—C32	−163.87 (12)
C5—C6—C8—C9	−155.55 (13)	C4—C5—C27—C32	67.74 (16)
O1—C8—C9—C14	−144.49 (14)	C32—C27—C28—C29	−0.3 (2)
C6—C8—C9—C14	31.6 (2)	C5—C27—C28—C29	−179.83 (14)
O1—C8—C9—C10	31.66 (19)	C27—C28—C29—C30	0.6 (2)
C6—C8—C9—C10	−152.29 (14)	C28—C29—C30—C31	−0.7 (2)
C14—C9—C10—C11	−2.0 (2)	C29—C30—C31—C32	0.6 (2)
C8—C9—C10—C11	−178.33 (14)	C30—C31—C32—C27	−0.3 (2)
C9—C10—C11—C12	1.4 (3)	C28—C27—C32—C31	0.2 (2)
C10—C11—C12—C13	0.2 (3)	C5—C27—C32—C31	179.72 (13)
C11—C12—C13—C14	−1.2 (2)	C6—C7—N1—C1	−148.49 (13)
C12—C13—C14—C9	0.6 (2)	C15—C7—N1—C1	31.67 (18)
C10—C9—C14—C13	1.0 (2)	C6—C7—N1—C3	0.4 (2)
C8—C9—C14—C13	177.12 (13)	C15—C7—N1—C3	−179.42 (11)
N1—C7—C15—C20	50.14 (18)	C2—C1—N1—C7	130.66 (13)
C6—C7—C15—C20	−129.69 (15)	C2—C1—N1—C3	−21.25 (14)
N1—C7—C15—C16	−130.46 (14)	N2—C3—N1—C7	−152.67 (12)
C6—C7—C15—C16	49.7 (2)	C21—C3—N1—C7	89.46 (14)
C20—C15—C16—C17	1.5 (2)	C4—C3—N1—C7	−36.33 (16)
C7—C15—C16—C17	−177.90 (14)	N2—C3—N1—C1	0.10 (14)
C15—C16—C17—C18	0.0 (3)	C21—C3—N1—C1	−117.77 (12)
C16—C17—C18—C19	−1.1 (3)	C4—C3—N1—C1	116.44 (12)
C17—C18—C19—C20	0.7 (3)	C1—C2—N2—C3	−35.78 (14)
C18—C19—C20—C15	0.9 (3)	N1—C3—N2—C2	22.05 (14)
C16—C15—C20—C19	−1.9 (2)	C21—C3—N2—C2	142.42 (11)
C7—C15—C20—C19	177.47 (14)	C4—C3—N2—C2	−92.91 (13)

### Hydrogen-bond geometry (Å, º)

*Cg*3 is the centroid of the C9–C14 phenyl ring.

**Table:**

*D*—H···*A*	*D*—H	H···*A*	*D*···*A*	*D*—H···*A*
C2—H2*B*···O1^i^	0.99	2.43	3.4084 (19)	168
C24—H24···*Cg*3^ii^	0.95	2.83	3.5886 (18)	138

Symmetry codes: (i) −*x*, −*y*, −*z*+2; (ii) −*x*, −*y*, −*z*+1.
